# A DNA Element Regulates Drug Tolerance and Withdrawal in Drosophila

**DOI:** 10.1371/journal.pone.0075549

**Published:** 2013-09-23

**Authors:** Xiaolei Li, Alfredo Ghezzi, Jascha B. Pohl, Arun Y. Bohm, Nigel S. Atkinson

**Affiliations:** The Waggoner Center for Alcohol and Addiction Research, Section of Neurobiology, the University of Texas at Austin, Austin, Texas, United States of America; University of Maryland School of Medicine, United States of America

## Abstract

Drug tolerance and withdrawal are insidious responses to drugs of abuse; the first increases drug consumption while the second punishes abstention. Drosophila generate functional tolerance to benzyl alcohol sedation by increasing neural expression of the *slo* BK-type Ca^2+^ activated K^+^ channel gene. After drug clearance this change produces a withdrawal phenotype—increased seizure susceptibility. The drug-induced histone modification profile identified the 6b element (60 nt) as a drug responsive element. Genomic deletion of 6b produces the allele, *slo* ^Δ6b^, that reacts more strongly to the drug with increased induction, a massive increase in the duration of tolerance, and an increase in the withdrawal phenotype yet does not alter other *slo*-dependent behaviors. The 6b element is a homeostatic regulator of BK channel gene expression and is the first cis-acting DNA element shown to specifically affect the duration of a drug action.

## Introduction

Drug tolerance and withdrawal symptoms are two of the seven criteria used to diagnose addiction in humans [1]. These two responses are central features of the addicted state and have long been proposed to be two faces of the same homeostatic response [2]. The very adaptations that generate functional tolerance, a reduction in the response to a drug caused by prior drug exposure, also generate an abnormal, typically opposite effect once the drug is cleared. For example, tolerance to a drug that suppresses neural activity could be produced by changes in gene expression that make the nervous system more excitable. However, when the drug is no longer present these changes produce an overly excitable nervous system. Such changes could underlie the long-recognized behavior of an addict or alcoholic who upon abstention begins to shake, is unable to sleep, and may even have seizures [3].

We use the Drosophila model system and the organic solvent anesthetic benzyl alcohol to understand the homeostatic regulatory mechanisms that produce functional tolerance to organic solvent sedation. Because of its low toxicity, benzyl alcohol has been used to model the neural effects of organic solvent exposure in the Drosophila model system [2]. Drosophila generate functional tolerance to sedation by the anesthetic benzyl alcohol by increasing neural expression of the *slo* BK channel gene [4]. By reducing the neural refractory period, the increase in *slo* expression acts as a neural excitant and counters the sedating effects of the drug [5]. While the change helps to counter the effects of the anesthetic, after drug clearance it produces a withdrawal phenotype by continuing to promote excitability, thus increasing the seizure susceptibility of the fly.

**Figure 1:**
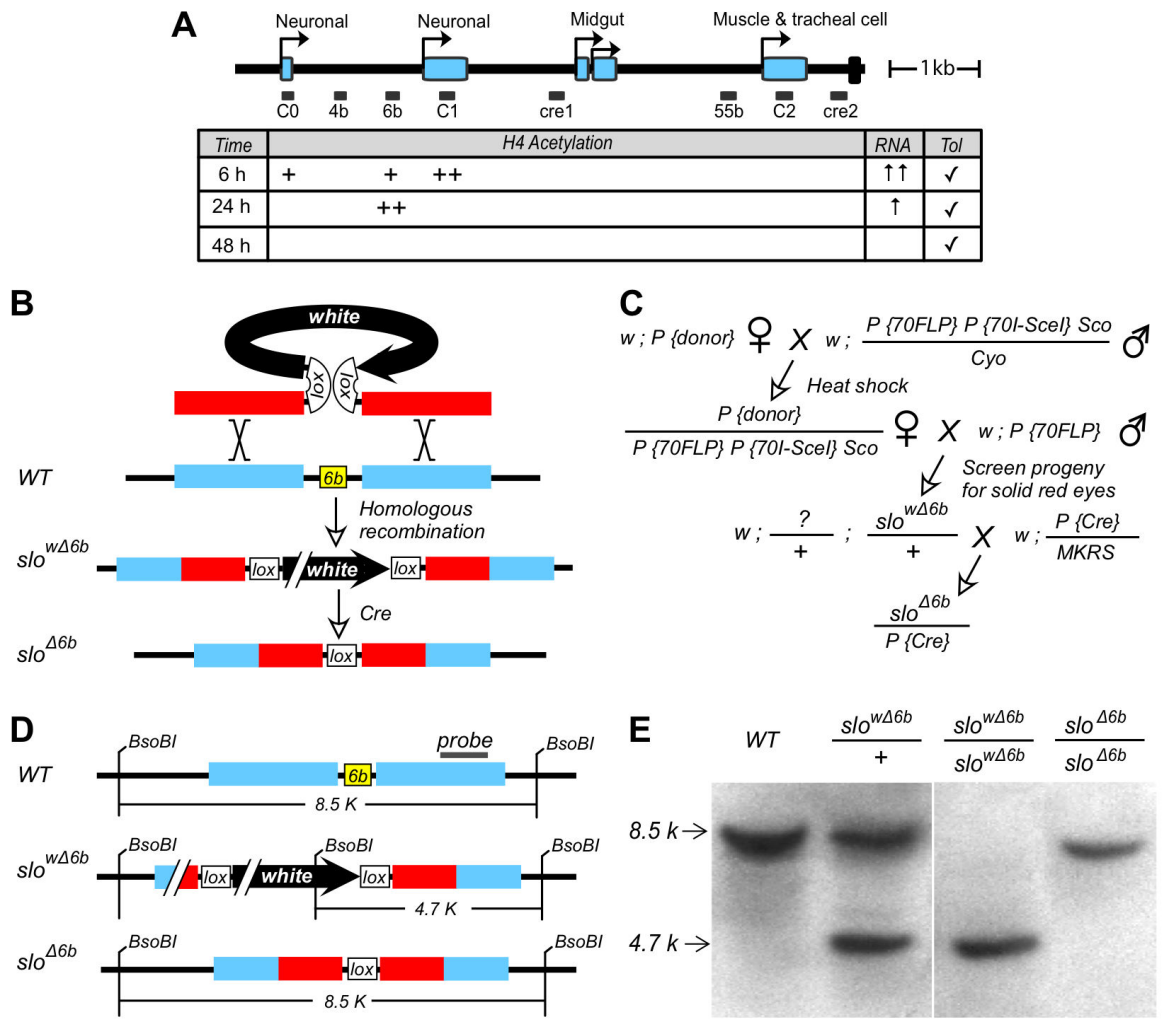
Modification of the *slo* transcriptional control region. **A**) Transcriptional control region of *slo*. Labelled arrows are tissue-specific transcription start sites of the previously-described promoters [11]. Blue boxes represent alternative 5' exons that are unique products of each promoter. Rightmost box on the line represents the first exon common to all *slo* transcripts. Neuronal splice variants begin translation in this exon. The remainder of the coding region is not shown. Boxes below the line are non-coding conserved DNA elements [11,40,41]. The table summarizes benzyl alcohol induced histone H4 hyper-acetylation, neural expression of *slo*, and functional tolerance (data from Wang et al. [6]). Plusses correspond to the H4 hyperacetylation. Arrows reflect relative abundance of *slo* mRNA (RNA). Checks identify when behavioral tolerance can be detected in the first 48 h (Tol). **B**) Homologous recombination occurred between the replacement DNA (red) and its chromosomal counterpart (blue) replaces 6b with a floxed mini-white gene. Cre recombinase was used to excise the loxP-flanked *white* gene to produce the *slo* ^∆6b^ allele in which the 6b element has been replaced by a loxP site. **C**) Crossing scheme for 6b targeting. **D**) Products produced at the different steps in the crossing scheme described in panel C**. E**) Southern blotting confirms homologous recombination into the *slo* locus. Restriction maps of wild type, *slo* ^w∆6b^ *, and* *slo* ^∆6b^. Probe indicated above maps. The recipient line (WT), produces an 8.5 kb band. The *slo* ^w∆6b^ and *slo* ^∆6b^ recombinants produce bands whose size is indicative of high fidelity homologous recombination into at the position of the 6b element (cf. panels D and E).

To determine how the transcriptional control region of the *slo* gene senses drug sedation, we previously mapped the position of a drug-induced histone modification under the assumption that these modifications mark sites of transcription factor activity and that the underlying DNA would contain functionally important DNA enhancers [6]. One such strong and focused drug-induced histone acetylation spike was situated between the two neural-specific core promoters and has been closely correlated with both drug-induction of *slo* expression and the appearance of behavioral tolerance. The DNA underlying this position includes a highly conserved 60 nt DNA element called element 6b (Figure 1A). This element was mutated *in situ* and shown to have a profound effect on the response to benzyl alcohol sedation.

## Results

The 6b evolutionarily conserved DNA element was previously proposed to be involved in benzyl alcohol regulation of the *slo* gene (reference [6] and summarized in Figure 1A). Because DNA regulatory elements can have context-specific effects we chose to delete the element from its chromosomal position and determine how its removal affects *slo* gene expression.

### Removal of the 6b element from the *slo* promoter region

Homologous recombination [7] was used to replace the sixty-nucleotide element 6b in the endogenous gene with a single loxP site (Figure 1B-C). Correct targeting events were verified by genetic linkage, Southern blotting (Figure 1D), and DNA sequencing. This allele, called *slo* ^∆6b^ was backcrossed into the wild-type Canton S background for six generations to minimize genetic differences between the mutant and wild-type lines. The *slo* ^∆6b^ homozygotes are healthy with no obvious physical abnormalities nor deficiencies in growth rate or development.

**Figure 2:**
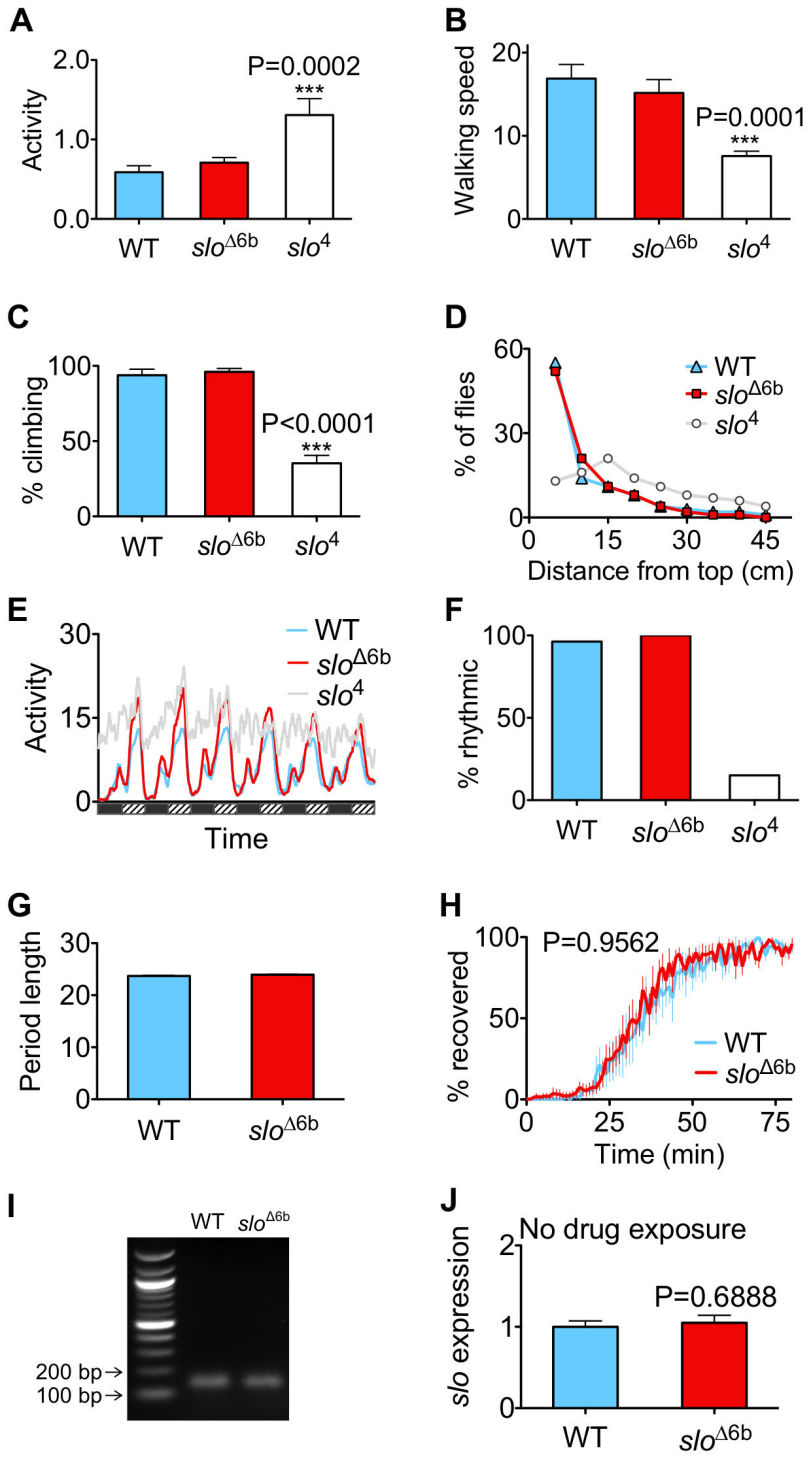
*slo* ^∆6b^ flies are normal with respect to most behaviors. The *slo* ^∆6b^ strain was backcrossed to the Canton S wild-type stock (WT) for six generations prior to analysis. The locomotor activity, (**A**), walking speed (**B**), climbing (**C**) and flight (**D**) of *slo* ^∆6b^ are similar to WT, while the *slo* ^4^ null mutants are deficient in all four behaviors. **E**) Circadian activity. LD entrained flies were transferred to DD and monitored. WT and *slo* ^∆6b^ flies demonstrated rhythmic oscillation in activity. *slo* ^∆6b^ had slightly higher peak activity than WT. However, the *slo* ^4^ null mutants were arrhythmic. n=25. **F**) Percentage of WT, *slo* ^∆6b^, and *slo* ^4^ identified as rhythmic. **G**) *slo* ^∆6b^ mutants had a normal circadian period length. n=25. **H**) The *slo* ^∆6b^ mutation does not alter drug resistance. Age-matched females were BA sedated and their recovery rate was determined. n=6. **I**) The *slo* ^∆6b^ mutation, does not disturb the splicing out of the intron in which it is located. RT-PCR shows that the flanking exons are spliced normally. **J**) The *slo* ^∆6b^ allele shows normal basal expression level (P=0.859; n=3). mRNA abundance was measured by RT-qPCR using primers specific for neuronal *slo* transcripts. **Statistical tests**. A, B, C, and F–one-way ANOVA with Dunnett’s comparison post test. *** indicates P≤ 0.001. N=25 (A), 3 (B), and 4 (C). For H, the log-rank test was used to evaluate significance between the recovery curves. For J, an unpaired *t*-test was used to evaluate significance. All error bars are SEM.

All of the original seven *slo* mutant alleles are believed to be null mutations, either failing to express protein or producing a protein that fails to assemble into a functional channel [8]. The null alleles were identified based on the so-called "sticky-feet" phenotype in which a short stimulation with heat followed by a return to ambient temperature causes the mutants to behave as if their feet are stuck to the surface upon which they stand [8,9]. In addition, without any sort of pretreatment, a *slo* null mutation also produces other subtle behavioral abnormalities. These include changes in overall activity, walking speed, climbing ability, capacity for flight, and circadian rhythmicity (shown in Figure 2 for the *slo* ^4^ null allele; some of these behavioral changes have been previously described [9,10]). Homozygous *slo* ^∆6b^ mutants do not show these phenotypes. As displayed in Figure 2A-G, homozygous *slo* ^∆6b^ mutants are indistinguishable from wild type for these behaviors. The *slo* ^∆6b^ allele also shows normal intrinsic sensitivity to sedation with benzyl alcohol (Figure 2H).

### *slo* ^∆6b^ mutants transcriptionally overreact to drug sedation

In the fly, neural expression of *slo* is the product of two core promoters C0 and C1 (Figure 1) [11]. Transcripts from core promoter C0 begin with a 37 nt exon that has been called exon C0, while transcripts from core promoter C1 begin with a 433 nt exon called exon C1. In the pre-mRNA, exon C0 is spliced to exon C1 [11]. Thus, quantifying the relative abundance of exon C1 in the mRNA pool reports the neural-specific expression level of the *slo* gene although it does not report the relative contribution of the two neural-specific core promoters. Bohm et al. [11] used RNAase protection assays to show that the ratio of C0 to C1 transcripts is 12:1. Because the *slo* ^∆6b^ mutation is within the intronic region separating exon C0 and exon C1, we were concerned that the mutation might interfere with the splicing of exon C0 to exon C1. To evaluate this possibility, we performed RT-PCR using a 5' PCR primer within exon C0 and a 3' PCR primer within exon C1. Amplification with this primer pair produced a product of the predicted size for a properly spliced mRNA, demonstrating that *slo* ^∆6b^ does not completely disrupt splicing of the C0-specific transcript (Figure 2I). However, it is possible that a small change in splicing efficacy has occurred.

**Figure 3:**
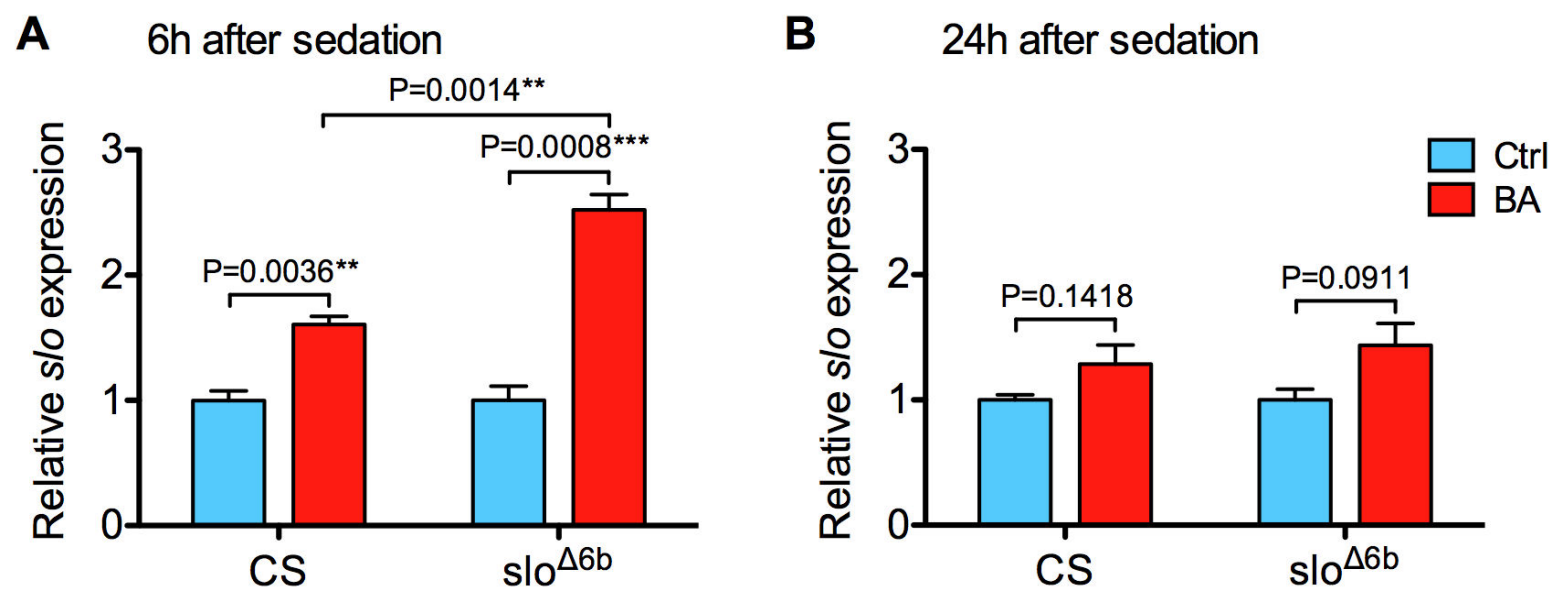
The *slo* ^∆6b^ mutation specifically affects drug-induced neural expression of *slo*. **A**) Six hours after benzyl alcohol sedation induction slo expression is increased in the wild type (CS) and *slo* ^∆6b^ mutants; however, induction is greater in the mutant than in the wild type. **B**) The relative abundance of *slo* mRNA was not statistically different from the baseline abundance 24 h after sedation for either the wild type or the *slo* ^∆6b^ mutant. Unpaired *t*-test, n=3. Error bars represent SEM.

We used RT-qPCR (reverse-transcription quantitative PCR) to quantify the relative abundance of *slo* mRNA in head-specific mRNA expressed from the neural promoters of the mutant before and after benzyl alcohol sedation. In animals that had never been sedated, the mutant displayed the same relative abundance of neural *slo* mRNA as did the wild type (Figure 2J), indicating that removal of the 6b element did not affect the basal rate of *slo* transcription. However, 6 hours after benzyl alcohol sedation, *slo* mRNA abundance was induced to a substantially higher level in the mutant than in the wild type (150% vs. 60% induction, respectively; Figure 3A,B). However, the duration of induction appears similar. At 24 h post sedation the mRNA abundance of both the wild type and the mutant are not statistically different from the baseline (Figure 3C,D).

### *slo* ^∆6b^ mutants acquire abnormally prolonged tolerance

**Figure 4:**
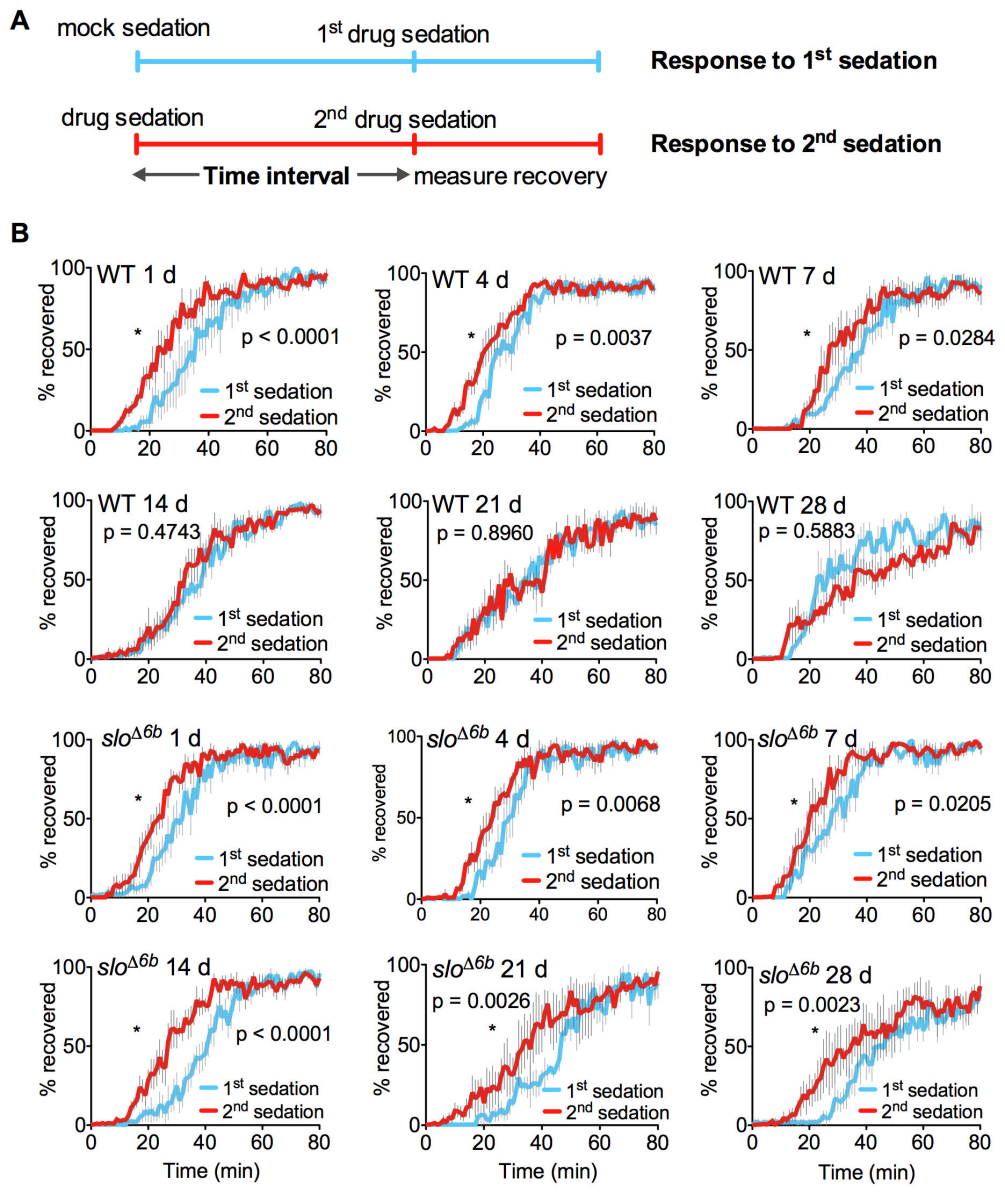
The *slo* ^∆6b^ mutant shows unusually long-lived benzyl alcohol tolerance. **A**) Schematic of the paradigm used to determine the time course of functional tolerance. A stock shows tolerance if it recovers more rapidly from its second sedation than from its first sedation. A population of age-matched females were separated into two groups. One group was mock sedated and the second group was sedated with benzyl alcohol. The animals were then housed in separate vials with food for the time intervals in panel B (1d -28 d) and then both groups were benzyl alcohol sedated in tandem, moved to fresh air (t=0), and their recovery recorded. **B**) The recovery curves describe the percentage of flies recovering after a single sedation (blue) and after a two sequential sedations (red) that were separated by the time interval shown. Tolerance lasts for at least 28 d in *slo* ^∆6b^; however, it is detected in wild type for only a week. Error bars represent SEM, but significance difference between curves is determined by log-rank analysis (n=4–6. *P ≤ 0.05).

One would expect that an increase in *slo* induction would result in a corresponding increase in the magnitude or duration of functional tolerance. While we did not observe an increase in the magnitude of 24 h tolerance, this might be because the response has saturated or because the assay is not well-suited for capturing the absolute magnitude of tolerance [4]. However, the mutation was observed to produce a profound difference in the duration of tolerance (Figure 4). In the time-course assay, we varied the interval between the first drug sedation and the tolerance assay from 1 day to 4, 7, 14, 21, and 28 days. Tolerance in the wild type persisted for less than 14 days, whereas the mutant still showed strong tolerance even 28 days after a single drug exposure. In fact, at the 28-day time point, while the mutant still showed substantial tolerance, the wild type exhibited the opposite response: sensitization to drug sedation. Tolerance was not assayed after 28 days because aging and mortality compromised the assay for both wild type and mutant. Recombinants that retained the 6b element but that carried an adjacent loxP insertion did not produce prolonged tolerance. In addition, loxP sequences have not been reported to be transcriptionally active. Therefore, we conclude that the increase in the duration of tolerance is caused by the absence of the sixty-nucleotide 6b element and not the presence of the loxP site.

**Figure 5:**
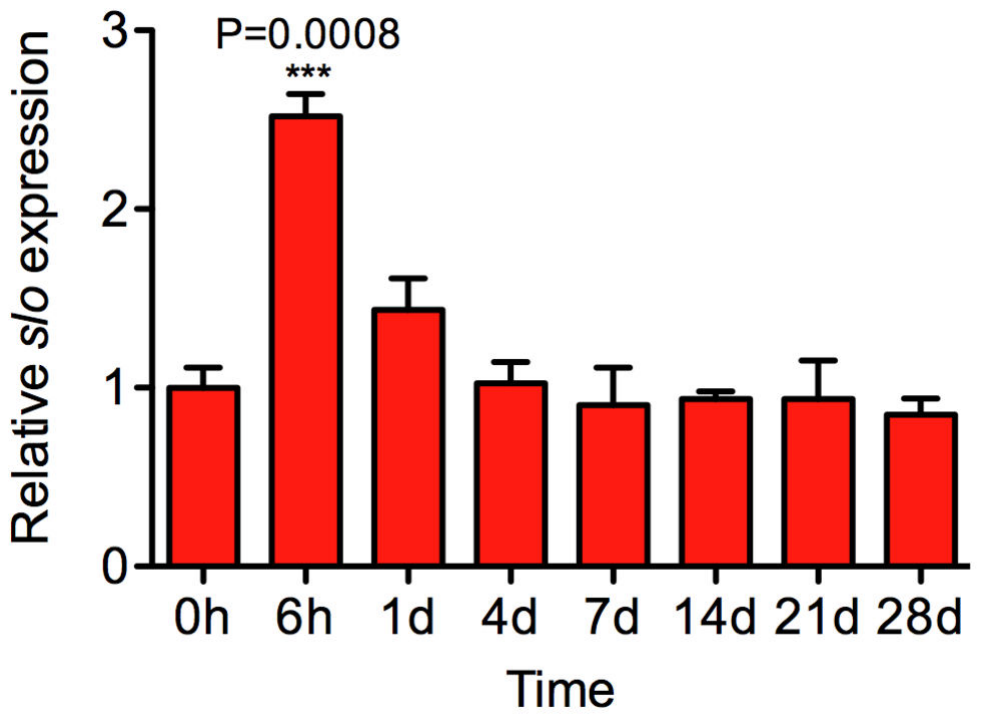
The time course analysis of *slo* transcription in *slo* ^∆6b^ flies. The mRNA levels of *slo* were determined by real-time RT-PCR using C1 primers that amplify only neural *slo* transcripts. One-way ANOVA with Dunnett’s comparison post test, n=3. Error bars represent SEM.

Prolonged tolerance might be caused by a pulsatile reactivation of the gene that maintains an elevated level of BK channel activity. However, a time-course analysis that monitored *slo* mRNA abundance in the nervous system from 6 h to 28 d after a single benzyl alcohol sedation (6 h, 1 d, 4 d, 7 d, 14 d, 21 d and 28 d) did not show evidence of an additional round of *slo* induction (Figure 5). It appears that the abnormal longevity of tolerance is the product of a single burst of *slo* expression. However, we cannot rule out the possibility that elevated *slo* expression is maintained in a minor but critical populations of neurons.

**Figure 6:**
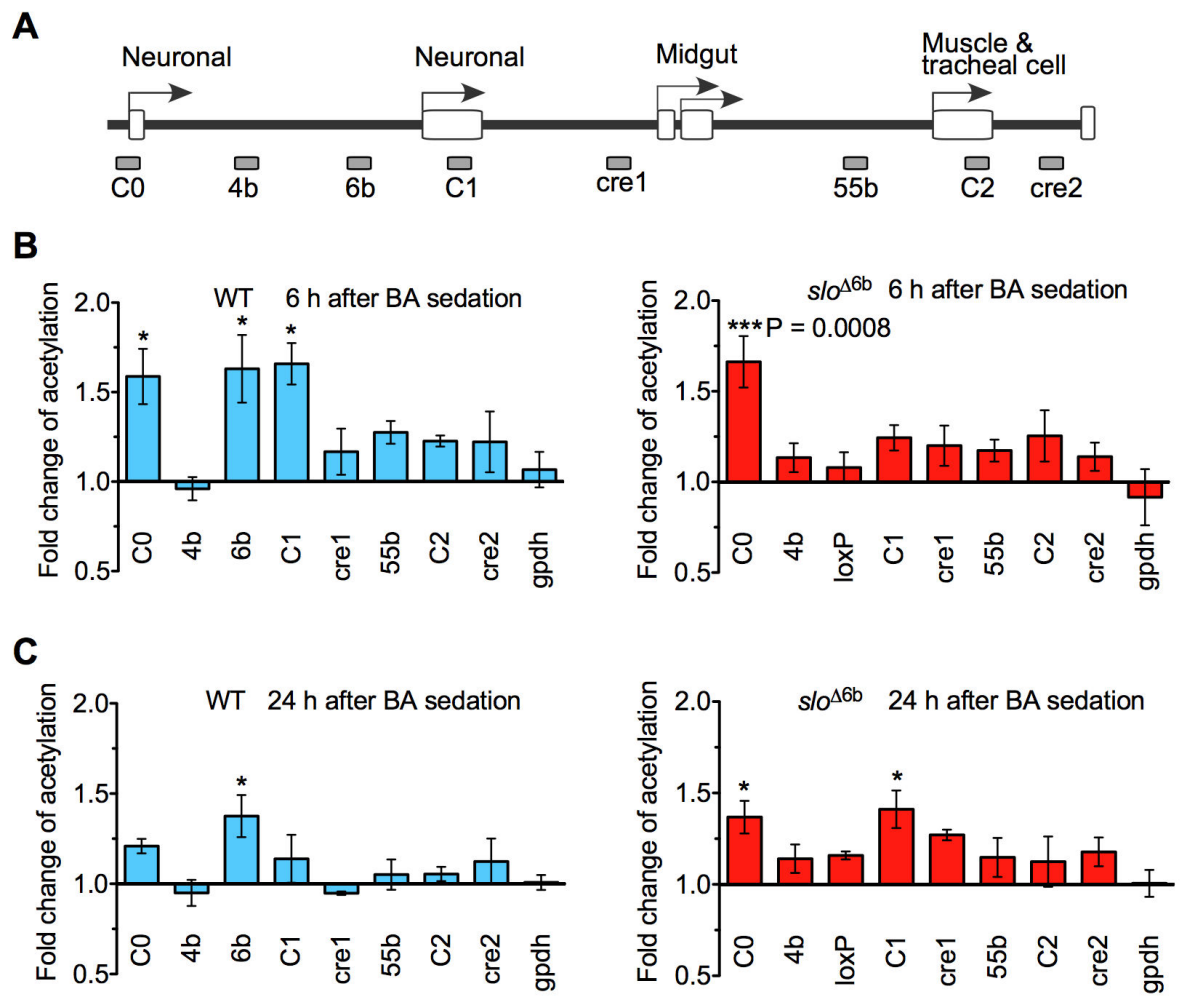
State of histone H4 acetylation across the *slo* transcriptional control region after benzyl alcohol sedation. **A**) Map of the *slo* transcriptional control region and areas assayed by the chromatin immunoprecipitation assay. Arrowheads identify the position of the tissue-specific *slo* core promoters, and open boxes on the line represent exons. The gray boxes below the line show the conserved elements tested in the chromatin immunoprecipitation assay. In *slo* ^∆6b^, the 6b site was replaced by a loxP element. **B**) H4 acetylation levels 6 h detected in WT and *slo* ^∆6b^ after benzyl alcohol sedation. One-way ANOVA with Dunnett’s comparison post test. n=3. From left to right * signifies P=0.0458, 0.0278, and 0.0200. *** signifies P=0.0008. Fold change of acetylation was the ratio of the acetylation levels of drug-sedated flies over untreated ones. **C**) Acetylation state surveyed 24 h after BA sedation. One-way ANOVA with Dunnett’s comparison post test. n=3. From left to right * signifies P=0.0387, 0.0486, and 0.0239. Error bars represent SEM.

Histone acetylation by transcription factors is a common step in gene activation and plays an important role in drug addiction [12]. Previously, we surveyed the histone H4 acetylation state to monitor the unfolding transcriptional program induced by benzyl alcohol sedation and identified element 6b as the location of a hyperacetylated spike observable 24 h after sedation [6]. There are also histone acetylation spikes over C1 and C0 at 6 h but not 24 h after sedation. The spike at C1 is probably merely part of the 6b spike since these two regions are contiguous. However, the spike at C0 is not continuous with 6b and probably is connected to transcription from the neural-specific promoter C0. Here we examined the drug-induced histone acetylation pattern in *slo* ^∆6b^ to ask how the loss of the 6b element affects this response. The histone acetylation pattern was assayed in a chromatin immunoprecipitation real-time PCR assay (ChIP-qPCR) using an antibody that recognizes all forms of histone H4 acetylation. At 6 h post sedation, the mutant lacks the wild-type acetylation spikes across the original position of element 6b and core promoter C1 although it shows strong acetylation at core promoter C0. At 24 h post sedation, the mutant lacks the acetylation spike at the position of 6b and instead shows significantly elevated acetylation at the two neural-specific promoters (core promoter C0 and core promoter C1; Figure 6).

### The *slo* ^∆6b^ mutation increases the physiological response to drug sedation

**Figure 7:**
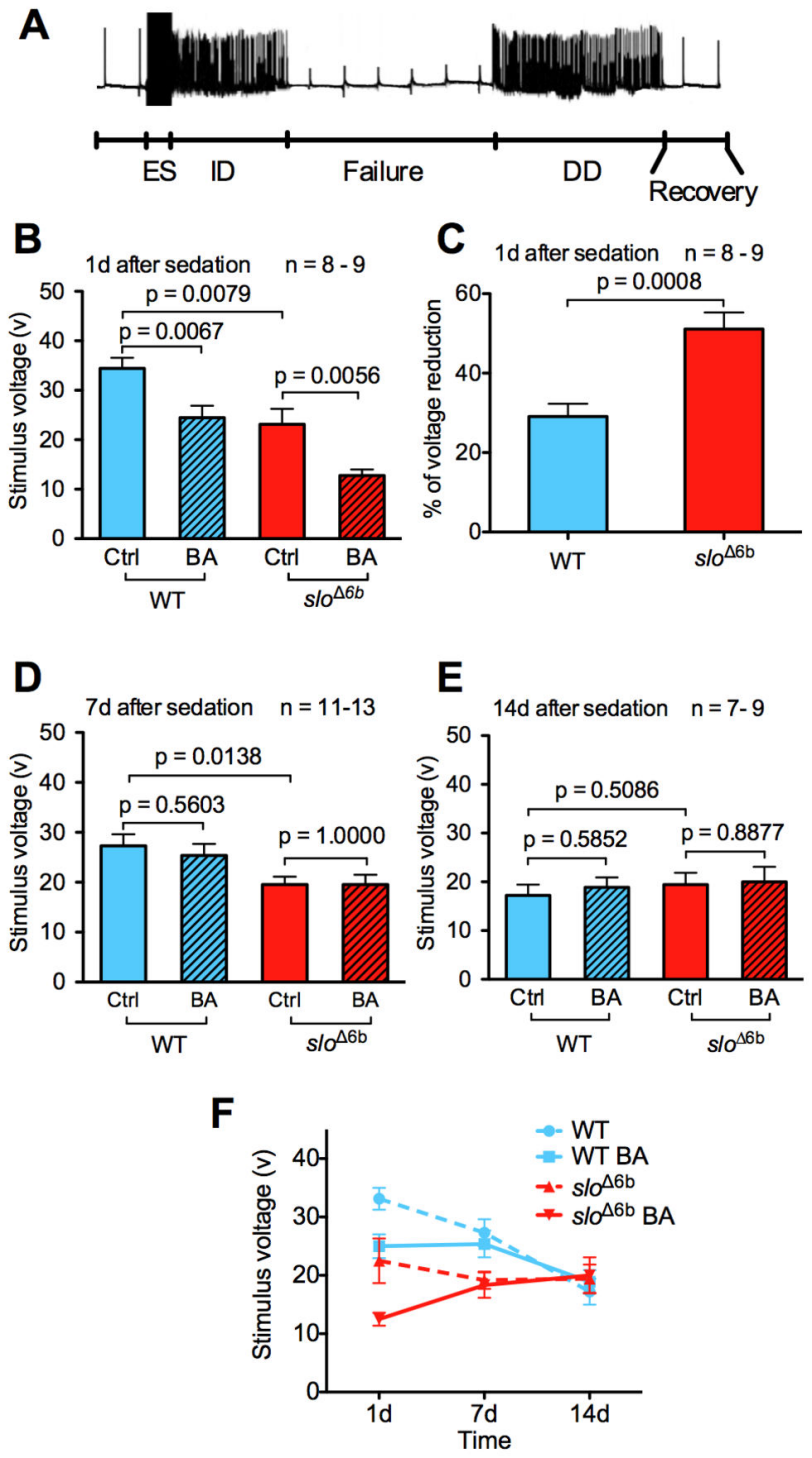
Electrophysiological analysis of wild type and *slo* ^∆6b^ stocks. **A**) Electroconvulsive stimuli (ES) induce a stereotypical seizure response from the giant fiber pathway. Constant low-frequency stimulations were applied continuously to assess the state of responsivity of the giant fiber pathway. A seizure consists of a high-frequency initial discharge (ID) followed by a period in which the giant fiber fails to respond to stimulation (Failure) followed by a delayed discharge (DD) and then by a recovery of normal responsivity. The seizure threshold was identified by delivering electroconvulsive shock of varying voltage until the production of a stereotypical seizure response. **B**) The *slo* ^∆6b^ mutant exhibited a significant lower average seizure threshold compared to wild-type flies. Benzyl alcohol sedation reduced stimulus voltage in both stocks. **C**) The drug-induced reduction in seizure threshold was greater in *slo* ^∆6b^ mutants than in wild-type animals one day after sedation. **D**,**E**) The average seizure stimulus voltages of both WT and *slo* ^∆6b^ returned to baseline at 7 d after sedation. **F**) Time course of seizure threshold after drug sedation. Re-plot of the data presented in panels B-E to illustrate the convergence of seizure threshold as a function of age. Wild-type (WT) and *slo* ^∆6b^ lines are compared before and after benzyl alcohol (BA) sedation. Unpaired Student’s t-test, P values and number of repeats as shown. Error bars show SEM.

Increased *slo* BK channel gene expression has been shown to act as a neural excitant that both counters the effects of organic solvent sedation, producing tolerance, and increases the probability of seizures after sedation [5]. In comparison to the wild type, *slo* ^∆6b^ mutants show increased *slo* induction following drug sedation and a concomitant increase in the duration of drug tolerance. Therefore, we asked whether the *slo* ^∆6b^ mutation would also exacerbate the post-sedation susceptibility to seizure phenotype. Trains of high-frequency electroconvulsive shock (ECS) delivered at escalating voltages were used to induce a stereotypical seizure response (Figure 7A) that is characterized by a spontaneous initial discharge followed by a period in which the neurons fail to respond and a delayed secondary discharge [13,14]. The seizure threshold voltage has been used as a measure of seizure susceptibility and is defined as the lowest stimulus voltage needed to induce a seizure.

Before sedation, the baseline seizure threshold was 34.4 ± 2.1 V in the wild type and 23.1 ± 3.1 V in *slo* ^∆6b^. This is the first indication of a change in a baseline response caused by the *slo* ^∆6b^ mutation. Twenty-four hours after benzyl alcohol sedation, the mutant showed a larger drug-induced change than the wild type showed. After benzyl alcohol sedation, the seizure threshold potential of the wild type was reduced from 34.4 ± 2.1 V to 24.4 ± 2.4 V, while the mutant showed a reduction from 23.1 ± 3.1 V to 12.8 ± 1.2 V (Figure 7B). To better compare the drug-induced response, the percentage decrease in seizure threshold was calculated by normalizing the voltage reduction by the basal seizure threshold voltage. Wild-type flies showed a drug-induced reduction of 29.0 ± 3.2% in seizure threshold, whereas the percentage change in seizure threshold in *slo* ^∆6b^ was 51.0 ± 4.2% (Figure 7C). The seizure threshold of both wild type and mutant had returned to baseline by 7 d post-drug sedation (Figure 7D& E). In addition, at the 7 d and 14 d time points we noted that there was an age-related reduction in seizure threshold in the wild type and that by 14 d post sedation all of the seizure thresholds had converged (Figure 7F).

## Discussion

This work represents the unusual discovery of a cis-acting DNA regulatory element that specifically participates in a complex behavioral response to a drug. The *slo* gene expresses BK-type Ca^2+^ activated K^+^ channels in most neurons of the CNS [15]. In accordance with this broad expression pattern, loss-of-function mutations in *slo* are behaviorally pleiotropic. These animals are uncoordinated, fly poorly, lack circadian rhythms, exhibit sticky-feet paralysis, are slightly resistant to seizures, and fail to acquire rapid tolerance to sedation with organic solvents such as benzyl alcohol and ethanol [4,9,10,16]. Sedation of flies with organic solvents was shown to homeostatically induce expression of the *slo* gene, which generates tolerance to sedation by these drugs and a withdrawal response [4,17]. The 6b element, positioned between the two neural core promoters of the *slo* gene, appears to be specifically involved in the drug-induced homeostatic regulation of neural expression. To date, the only behaviors observed to be affected by removal of the 6b element are drug-related responses—functional drug tolerance and drug-induced increase in seizure susceptibility. The effect of the mutation on basal expression from the gene appears to be minimal because the mutant animal is largely behaviorally normal and because *slo* ^∆6b^ homozygotes have the same relative abundance of neural-specific *slo* transcripts.

With regard to drug responses, the *slo* ^∆6b^ allele behaves as a gain-of-function mutation. Wild-type animals respond to benzyl alcohol sedation with an increase in *slo* neural expression that has been directly linked to the production of functional tolerance to sedation [4]. The *slo* ^∆6b^ mutant overreacts to drug sedation by producing an increased induction of *slo* expression without changing the duration of induction. This molecular overreaction appears to precipitate a rather substantial increase in the behavioral response. The mutation vastly exaggerates the duration of functional tolerance from ~10 days to at least 28 days. Technical limitations prevent us from assaying tolerance after this point, therefore twenty-eight days is the lower limit on the duration of tolerance in the mutant.

The extra BK channels produced as a consequence of *slo* induction could outlive the transient increase in *slo* message abundance. However, it seems improbable that the mutant allele could produce a sufficient superabundance of BK channels in 24 h to maintain tolerance for the full 28 days. In concordance with this supposition, the duration of the increase in seizure susceptibility also suggests that the *slo* ^∆6b^ allele does not cause a 28 d pan-neural increase in BK channel density after drug sedation.

Drug-withdrawal phenotypes and functional tolerance have been long proposed to have a common origin. This relationship was first described in the homeostatic theory of tolerance and dependence described by Himmelsbach [18] and refined by Martin [19]. The idea here is that the same changes that produce functional tolerance persist after drug clearance, temporarily unbalancing the nervous system, to cause the withdrawal symptom. Intrinsic to this idea is that the withdrawal symptom is expected to be opposite to the effect of a drug. Thus, a drug that depresses neural excitability is proposed to trigger pro-excitatory changes that counter the effects of the drug and result in tolerance. Once the drug is cleared, the excitatory changes will be uncovered resulting in a hyperexcitable nervous system. While this hypothesis probably does not hold true for all drugs, in flies, the relationship of the *slo* gene to benzyl alcohol functional tolerance and the benzyl alcohol withdrawal symptom (lowered seizure threshold) are in concert with the predictions of the hypothesis. In response to benzyl alcohol sedation, the *slo* gene is induced with pro-excitatory consequences (possible mechanisms described below) and this shortens the duration of sedation by benzyl alcohol [4]. The next day, long after benzyl alcohol clearance, the excitatory change produced by *slo* induction persists and in the absence of the drug causes the nervous system to be hyperexcitable [5,20].

How can an increase in the expression of the *slo* BK channel gene act as a neural excitant? The dogmatic view is that increases in K^+^ channel activity always produces a decrease in neural excitability and certainly, in some cells BK channel activity reduces excitability [21]. However, it has also long been known that in some cells BK channels have the opposite relationship to excitability. It was first noted by Warbington et al. [22] that, in flies, eliminating BK channel expression reduced neurotransmitter release at the larval neuromuscular junction. In mammals, BK channels are also the product of the *slo* gene. In pituitary cells, BK channel activity was shown to have a positive rather than a negative relationship to repetitive firing [23,24]. This positive relationship between channel activity and cellular excitability has also been documented in the mammalian CNS. In the dentate gyrus, BK channel activity increases the capacity for repetitive firing and a gain-of-function mutation in the human *slo* gene even causes an epilepsy [25-27]. In all of these mammalian examples, increased BK channel activity causes the action potential to repolarize more rapidly. This change prevents the inactivation of voltage-gated Na^+^ channels, or the activation of other K^+^ channels (e.g. SK channel) that would produce a long lasting after-depolarization—either of which extends the refractory period of the neuron. Thus, increasing BK channel activity increases net neuronal excitability by shortening the refractory period.

The same positive correlation between *slo* BK channels and neural excitability exists in flies. Ghezzi et al. [5] showed that transgenic over expression of the *slo* BK channel gene shortens the refractory period of the giant fiber pathway of Drosophila and lowers the seizure threshold of the fly whereas blocking *slo* expression was shown to increase the seizure threshold [28]. The increased susceptibility to seizures is a withdrawal phenotype because it is precipitated by a drug but is only apparent after drug clearance. A similar withdrawal phenotype has been observed for flies in benzyl alcohol withdrawal and for flies in ethanol withdrawal [5,20].

BK channels expressed from the *slo* gene have also been shown to underlie a form of functional ethanol tolerance described in the rat hypothalamic-neurohypophysial explant system [30]. In this system, ethanol potentiates BK channel activity which leads to an increase in firing and a suppression of hormone release. However, chronic ethanol exposure leads to tolerance. Tolerance is produced by internalizing preexisting BK channels and replacing them with ethanol-resistant channels. The shift to the production of ethanol-resistant BK channels has been documented in both the hypothalamo-neurohypophysial and medium spiny neurons of rats and is caused by the activation of a microRNA (mir9) that targets *slo* transcripts encoding ethanol-sensitive channels [31].

In mammalian model systems of *slo*-dependent ethanol tolerance, transcriptional regulation has not been identified as a key factor, nor has post-transcriptional regulation been identified as important in the Drosophila model of organic solvent tolerance. This may, however, be a product of the different properties and experimental advantages offered by each model system although it is also possible that different organisms emphasize different regulatory regimens to achieve related ends.

Because changes in *slo* expression underlie both functional tolerance and the reduced seizure threshold withdrawal phenotype, one would expect that the *slo* ^∆6b^ mutant would affect tolerance and the seizure threshold phenotype in the same way. While both responses are increased, it is the duration of the tolerance phenotype that increases, while it is the magnitude of the seizure phenotype that changes. Thus, in a sense they both change in the same direction, however it is not quite in the manner expected.

What might account for the difference in time course between the effect of the mutation on the tolerance and seizure phenotypes? Perhaps the amount of *slo* expression required to produce the tolerance phenotype is lower than the expression threshold required for the withdrawal phenotype. The turnover rate of BK channels might then account for the difference in duration of these two drug endophenotypes. An exponential decay of the extra BK channels could cause channel activity to fall below the threshold for seizure susceptibility before it drops below the threshold for tolerance. Thus, a single change in gene expression could produce both the relatively short change in seizure threshold and the long lasting change in functional tolerance. Alternatively, tolerance and the susceptibility to seizure withdrawal phenotype may arise from different neural structures with different turnover rates for the BK channel or even different *slo* induction dynamics after drug sedation. Perhaps the *slo* ^∆6b^ allele is persistently active in the structure underlying tolerance but only transiently active in the structure(s) responsible for the change in seizure threshold. Because RT-qPCR does not detect overexpression throughout the period of tolerance, this overexpressing structure would have to be small and/or the level of induction would have to be small. The lack of synchrony in the duration of tolerance and withdrawal phenotypes might also be the product of cellular- or system-level adaptions to increased BK channel activity that differentially affect these two phenotypes. Finally, the apparent disconnection between the duration of these phenotypes could merely be a side-effect of an age-related change. Indeed, in the wild type we observed an age-related reduction in seizure threshold (Figure 7F). It is possible that this reduction eventually obscures any drug-mediated reduction in seizure susceptibility.

The observation that the *slo* ^∆6b^ allele depresses the baseline seizure threshold means that the mutant must alter the expression pattern of *slo* even in the absence of benzyl alcohol exposure. However, if the alteration occurs in the adult then it must be subtle given that the change is not detectable by RT-qPCR. This might occur in multiple ways. The mutant allele could produce a tiny pan-neuronal change in expression that is sufficient to produce the phenotype. It could also be that the mutation alters expression in a specific but tiny part of the nervous system and this lowers the seizure threshold. Alternatively, the mutation may produce a change in expression during development that reduces the seizure threshold in the adult nervous system even though *slo* expression in the adult is normal.

The 6b element was identified as a candidate element because it underlies a drug-induced histone acetylation spike. In the wild type, increased histone acetylation persisted over 6b for 24 h after benzyl alcohol sedation. Furthermore, the HDAC inhibitor sodium butyrate induced *slo* expression, phenocopied tolerance, and generated a single acetylation spike over element 6b [6]. This led us to hypothesize that a histone deacetylase was chronically positioned at 6b and that perhaps the regulation of this deacetylase contributed to *slo* induction. The response of the *slo* ^∆6b^ mutants to benzyl alcohol sedation suggests that this interpretation is correct—that 6b is a negative regulator of *slo* expression, albeit it is possible that it is not the absence of 6b that produces the phenotype but instead small changes in the spacing between elements that flank 6b.

Examination of the 6b element shows that it contains transcription factor binding site motifs that might account for this mutant phenotype. Within 6b there is a heat shock element (HSE) motif that could be recognized by the heat-shock regulatory transcription factor (HSF) [6]. HSF has been shown to regulate histone acetylation levels in mammals through its association with HDAC1 and HDAC2 [29]. In a homologous manner a Drosophila HSF transcription factor might help to position HDACs at 6b. Deletion of the 6b element might permanently prevent the positioning of regulatory HDACs that are used to limit or terminate homeostatic activation of the *slo* gene, and thereby the mutant overresponds to induction by benzyl alcohol.

Histone modifications serve as molecular footprints that visualize the action of transcription factors. This study demonstrates the profound utility of epigenetic histone modifications to map the position of DNA elements that mediate specific regulatory responses. The map of the dynamic histone modification changes induced by drug exposure helped to narrow the search for a regulatory DNA element that mediated the drug-induced change in transcription. It is unlikely that the 6b element would have been identified as functionally interesting by any other method of sequence analysis.

## Materials and Methods

### Fly stocks

Drosophila stocks were Canton S (wild type), *slo* ^4^, and *slo* ^∆6b^. Flies were raised on standard cornmeal medium in a 12/12 light/dark cycle at 22°C. Newly eclosed flies collected during a 2 d time period were studied 4–5 days after eclosion.

### Ends-out gene targeting of the 6b element

The homologous recombination method of Gong and Golic [7] was used to precisely replace the ~60 nt 6b element with a 80 nt loxP sequence. The two flanking DNA fragments of the *slo* 6b element were amplified by PCR with Canton S genomic DNA template and proofreading *PfuTurbo* DNA polymerase (Stratagene CA) and inserted into the ends-out vector pW25 [32]. Primers 5'-gcggccgcgagggatgctgctgctttac-3' and 5'-gcggccgcattctcgccgtttttaaattctcag-3' were used in the PCR to amplify and add *Not*I sites to both ends of a 3.26 kb DNA fragment upstream of the 6b element, while primers 5'-ggcgcgccttgacacgcttgcttttccggcta-3' and 5'-ggcgcgccaagttggcccagtttgtttg-3' were designed to amplify and add *Asc*I termini to a 3.03 kb DNA fragment downstream of 6b. These two donor fragments were then inserted into the corresponding sites of pW25 with the same orientation. The 6b deletion construct was introduced into a *w* ^1118^ background by *P* element germ line transformation. The chromosomal insertion of the donor transgene was genetically mapped, and lines with the transgene on the first or the second chromosome were utilized to induce targeting on the third chromosome.

The scheme for targeted replacement of the 6b is shown in Figure 1. To induce targeting, donor insertion lines are crossed to the *P*{*hsp70FLP*} *P*{*hsp70I-SceI*} strain (Bloomington # 6934). The progeny were heat shocked during the first 3 days of development to induce FLP and *Sce*I expression. The heat shock was 38°C for 1 h. Females with donor and *P*{*hsp70FLP*} *P*{*hsp70I-SceI*} were crossed to male FLP flies (Bloomington # 6938), and the progeny were screened for solid red eyes. The transgenic *w* ^+^ gene was mapped to identify targeting events in which *w* ^+^ was relocated to the target chromosome 3. Finally, the targeted strain was crossed to *P*{*Cre*} line (Bloomington # 1501) to remove the *w* ^+^ marker and generate the 6b deletion mutant *slo* ^∆6b^. The sequence of the modified gene has the Genbank accession # KF514522.

### Southern blotting analysis

Southern blot analyses were performed using a DIG kit (Roche Diagnostics, IN). Genomic DNA was digested overnight with *Bso*BI (NEB Inc., MA) and subjected to agarose gel electrophoresis and nylon membrane transfer as described [33]. The DNA probe was a 0.6 kb fragment of the *slo* promoter region prepared by PCR with primers 5'-GCGACCACAAATCGTCCAAACACA-3' and 5'-TAGCACTGCGAACTATCGCTGGAA-3'. The blot was hybridized to the digoxigenin (DIG) labelled probe and visualized with DIG antibody-coupled chemiluminescence.

### Benzyl alcohol sedation

Benzyl alcohol sedation of ten 3–5 d old female flies was conducted as described [4]. Briefly, 30 ml glass vials were coated with 200 µl of 0.4% benzyl alcohol in acetone and then rotated constantly for 20 min to evaporate the more volatile acetone and leave a layer of benzyl alcohol on the wall. In contrast, the vials for controls were coated with acetone only, which was completely evaporated to dryness after 20 min. Animals were transferred into either the benzyl alcohol vials or the control vials until all the flies in the drug vials were completely sedated (usually 5-10 min). After exposure, flies were moved back to food vials for recovery.

### Tolerance assay

The capacity to acquire tolerance was measured as described [4]. Recently eclosed females were divided into groups of 10. In the first exposure, half were sedated with 0.4% BA, and half were mock sedated with acetone. On the day of assay, all groups were sedated with 0.4% BA and transferred to fresh vials to recover. Flies were scored as recovered when they resumed climbing. The whole course of recovery was analyzed by a computer-controlled automated movement detection system [34]. The recovery curves were plotted as the percentage of recovered flies against time.

### Chromatin immunoprecipitation (ChIP) assay

ChIP assays were performed using the Upstate/Millipore ChIP kit. Approximately 1,000 flies were either benzyl alcohol sedated or mock sedated for 6–8 minutes and then allowed to recover in a drug-free environment. Six hours and 24 h after sedation, flies were frozen in liquid nitrogen, and heads were harvested by vortex decapitation and sieving. Chromatin was prepared as described^5^, sheared to ~600 bp and precipitated using a polyclonal antibody against histone H4 acetylated at K5, K8, K12, and K16 (part 06-866, Upstate/Millipore) at 1:200 dilution. Total DNA was measured in one-tenth of the lysate for normalization purposes. ChIP assays were performed at least three times with independent chromatin samples.

Real-time PCR was performed using the ABI SYBR Green PCR Master Mix (Applied Biosystems) with an ABI 7300 real-time PCR system (Applied Biosystems). Primers were designed to amplify 100~200 bp fragments of the *slo* transcriptional control region, including the two neural promoters (C0, C1), the muscle promoter (C2), and five evolutionarily conserved regions (4b, 6b/loxP, cre1, 55b, and cre2). Genes *Gpdh* (Glycerol-3-phosphate dehydrogenase) and *Cyp1* (Cyclophilin 1) were used as internal controls because their transcription and acetylation levels are independent of benzyl alcohol sedation [6]. Primer sets are: C0 (5'-ATCGAACGAAGCGTCCAG-3', 5'-CGACGCGCTCAAACG-3'); 4b (5'-GACCCGATGATAAAGTCGATGT-3', 5'-GCCA GTGACTGACTGACACACA-3'); 6b (5'-CCAGCAGCAATTGTGAGAAA-3', 5'-CGAAGCAGACTTGAAAGCAA-3'); loxP (5'-AAACGGCGAGAATGCGGC-3', 5'-TCAAGGCGCGCCCTAGACT-3'); C1 (5'-ACAAACCAAAACGCACAATG-3', 5'-AATGGATGAAGGACTGGGAGT-3'); cre1 (5'-GATGGGAAAGCGAAAAGACAT-3', 5'-CATGTCCGTCAAAGCGAAAC-3'); 55b (5'-TACCCAATTGAATTCGCCTTGTC TT-3', 5'-CCCACTCTCCGGCCATCTCT-3'); C2 (5'-GCACTCGACTGCACTTGAAC -3', 5'-AATGAAAAAGTTCTCTCTGTGCAT-3'); cre2 (5'-TGGATTGCGACCGAGTG TCT-3', 5'-ATCAATACGATAACTGGCGGAAACA-3'); Gpdh (5'-GCATACCTTGATCTTGGCCGT-3', 5'-GCCCTGAAAAGTGCAAGAAG-3'); and Cyp1 (5'-TCTGCGTATGTGTGGCTCAT-3', 5'-TACAGAACTCGCGCATTCAC-3'). The PCR protocol began with a denaturation cycle of 95 °C/5 min, followed by 40 cycles of 95 °C/15 s, 60 °C/30 s, and ended with a single cycle of 72 °C/30 s. Real-time PCR amplifications were run in duplicate, and the dissociation/melting curves were used to detect nonspecific amplifications. The relative amount of the DNA precipitated by the H4Ac antibody was determined by ∆∆Ct method. Fold enrichment of H4 acetylation of the drug-sedated group equals 2^(∆Ct^ctrl^ - ∆Ct^BA^) where ∆Ct = Ct^IP^ - Ct^Input^.

### Reverse transcription-quantitative PCR analysis

RNA was extracted from heads as described [4]. Reverse transcription and real-time PCR were performed in triplicate with *slo* exon C0, C1, and *Cyclophilin 1* (*Cyp1*) primers [17]. Relative RNA quantity was calculated using the standard curve method. Forward and reverse primers were as follows: for *slo* exon C0, 5'-ATACGCTGCTGACGAGAAAGGTTG-3' and 5'-ACTGCGCTTAGTCACACTGCTCAT-3'; for *slo* exon C1, 5'-AAACAAAGCTAAATAAGTTGTGAAAGGA-3' and 5'-GATAGTTGTTCGTTCTTTTGAATTTGA-3'; and for *Cyp1*, 5'-ACCAACCACAACGGCACTG-3 and 5'-TGCTTCAGCTCGAAGTTCTCATC-3'.

### Walking and climbing assay

Walking speed was measured with females in a 12-well (35 mm x 5 mm) culture plate. A single fly was placed in each well and allowed 5 minutes to equilibrate, and then activity was recorded in a 10 min video recorded with a webcam. Eight 4 s videos clips of walking flies were manually measured to determine the distance traversed.

The climbing assay was conducted as described to measure relative activity of flies [35]. Three groups of 12 flies each were placed in a 20 mm × 150 mm glass test tube and gently tapped to the bottom. The number of flies that climbed beyond 10 cm within 10 sec was counted.

### Flight assay

The flight capacity of animals was determined as previously described for *slo* mutants [36]. Approximately 500 flies were dropped into the center of a mineral oil–coated 15 cm × 62 cm pipette jar through a funnel at the top. The falling animals fly from the center and are trapped in the oil. The distance that they fall is correlated with the capacity for flight. The position of each fly was marked and its distance from the top was measured. Flies with good flight capacity clustered near the top of the jar, whereas flies that flew poorly stayed close to the bottom.

### Sticky-feet behavioral assay

The sticky-feet behavioral test was performed as described [36]. Briefly, flies were heat-shocked in a glass vial at 37°C for 7 min. Animals were then transferred to the benchtop and left undisturbed for about 10 sec. A flat toothpick was used to push on the sides of the flies. Sticky-feet is defined as when a fly hangs onto the surface instead of flying or walking away.

### Circadian rhythm assay

Flies were raised in a rhythmic 12:12 light:dark cycle. Males were individually loaded, without anesthesia, into 5 mm × 65 mm glass tubes with 5% sucrose 2% agar food at one end. These tubes were then loaded into Drosophila Activity Monitors (DAM2, Trikinetics, Waltham MA) that detect movement using infrared sensors. The monitors were placed into a sound-isolated incubator at 24°C, and free-running rhythms were measured in constant darkness in 5 minute bins for 14 days. Any fly that did not move for any 24 hour period in the first ten days was discarded from analysis. Period and rhythm indices were generated by the autocorrelation as described [37]. Rhythmic animals were those with a rhythm index greater than 0.1 [38]. Average daily activity was determined by summing and then averaging the number of DAM2 beam passes over a 7 day period.

### Seizure susceptibility analysis

Seizure susceptibility was measured by determining the seizure threshold of the giant fiber pathway as described [5]. Briefly, a female was mounted on a stereoscope stage in a small mount of paraffin wax. Two stimulating 200 µm diameter sharpened tungsten wire electrodes (FHC Inc.) were inserted through the anterior face of the head on each side, between the compound eyes and the antennal sockets, halfway through the head into the brain of the fly using micromanipulators (Narishige, Inc.). Similarly, a sharpened 75 µm diameter recording electrode was inserted into the upper most DLM flight muscle through the dorsal thoracic cuticle while a sharpened 200 µm diameter reference electrode was placed in the abdomen. Stimuli were generated using a S48 square pulse stimulator, isolated with a SIU5 Stimulus Isolation Unit (Grass-Telefactor). Responses from the DLM were amplified with a Microelectrode Amplifier Model 1800 (A-M Systems) and digitized by a DigiData 1200 (Axon instruments). Data were recorded and analyzed in a PC using FETCHEX pCLAMP 6 software (Axon instruments).

Low-frequency (0.2 Hz) stimulating 30 V pulses were applied to the brain continuously to assess the health of the giant-fiber pathway. Successful responses to each stimulus pulse were detected at the DLM by the recording electrode as sharp action potential-like depolarizations. For each fly, seizures were triggered by 1.5 s long 200 Hz electro-convulsive shock (ECS) pulses of increasing voltage at 10 V steps in 8-minute intervals until a stereotypical seizure was elicited (Figure 6A).

### Statistical analysis

In the tolerance assay, the log-rank test for equality of survival was used to determine the significance between sedation recovery curves [39]. This statistic is well-suited for evaluating differences in the time to an event (such as recovery). The statistic evaluates whether entire recovery curves are different and not whether differences exist at any single time point.

In the ChIP analysis, the entire protocol was repeated at least three times, and significance was determined by one-way ANOVA. For RT-qPCR (n=3) and for changes in seizure threshold (n=5-9), significance was determined using an unpaired Student’s *t* test. Statistical significance in the daily activity (n=25), walking speed (n=3), and climbing assay (n=4) were determined by one-way ANOVA and Dunnett’s post-hoc analysis.
